# Efficacy and safety of Xuanfei Baidu granules for treating COVID-19: A protocol for systematic review and meta-analysis

**DOI:** 10.1097/MD.0000000000025653

**Published:** 2021-05-21

**Authors:** Jisen Zhao, Dong Guo, Maoxia Fan, Yongcheng Liu

**Affiliations:** a The First Clinical Medical College of Shandong University of Traditional Chinese Medicine; b Teacher Development Center of Shandong University of Traditional Chinese Medicine; c Shandong Provincial Hospital of Traditional Chinese Medicine, Jinan, Shandong Province, China.

**Keywords:** corona virus disease 2019, meta-analysis, protocol, systematic review, Xuanfei Baidu granule

## Abstract

**Background:**

Corona Virus Disease 2019 (COVID-19) is currently prevalent in most countries around the world. It has become a common threat to global human health because there is no specific cure and no targeted treatment for this disease at this stage. Xuanfei Baidu granule (XFBD) included the traditional Chinese medicine prescription in COVID-19 diagnosis and treatment Plan (trial eighth Edition) released in August 2020, which has played a great role in the diagnosis and treatment of COVID-19 in Wuhan, China. This paper intends to evaluate the efficacy and safety of Xuanfei Baidu granule in the treatment of COVID-19.

**Methods:**

The search strategies of different websites were searched on Cochrane Central controlled Trials Registry, PubMed, excerpt database, Web of science, China National knowledge Infrastructure, Chinese Science and Technology Journal Database, WanFang and other websites. All qualified studies were confirmed to include randomized controlled trials. The search time range was from January 1, 2019 to February 28, 2021. In the meanwhile, the list of references and related reviews was checked. Two evaluators were responsible for the extraction and management of the data independently. The literature quality was evaluated according to Cochrane manual 4.2.2. Heterogeneity test and Meta analysis were carried out by Review Manager V.5.3 software. The bias risk included in the study was evaluated by Cochrane “bias risk” tool, and the relevant statistical data were evaluated by GRADE3.6 evidence quality grading system.

**Results:**

This study intends to evaluate the efficacy and safety of XFBD in the treatment of COVID-19 from 4 aspects, including nucleic acid negative conversion time, average hospital stay, clinical symptom improvement rate and lung computed tomography improvement rate.

**Conclusion:**

The conclusion of this scheme intends to provide evidence for judging whether the intervention of XFBD on COVID-19 patients is effective or not.

**PROSPERO registration number:**

CRD42021245640

## 1 Introduction

The epidemic of Corona Virus Disease 2019 (COVID-19) appeared in Wuhan, China at the end of December 2019,^[1]^ and then quickly spread to other areas in China. On March 11, 2020, the World Health Organization declared COVID-19 a global pandemic.^[2]^ Since then, it has become a major global public health problem in the 21st century. Up to March 29, 2021, SARS-CoV-2 has caused 127832853 infections and 2797203 deaths, and the cumulative number of confirmed cases and deaths is still on the rise according to the Johns Hopkins Coronavirus Resource Center. COVID-19 is caused by severe acute respiratory syndrome coronavirus type 2 (SARS-CoV-2).^[3]^ The early symptoms of COVID-19 include fever, cough and fatigue. Some patients are mainly characterized by gastrointestinal symptoms, such as vomiting and diarrhea. A week later, the patients developed chest tightness, dyspnea and respiratory distress, and some patients rapidly developed into acute respiratory distress syndrome ((ARDS)) and septic shock, and even died.^[4]^ The late stage of COVID-19 can cause serious damage to a variety of organ functions of patients. According to a retrospective study of COVID-19 in critically ill patients, 67.3% of patients with ARDS,28.9% had acute renal injury, heart injury accounted for 23.1%, and abnormal liver function accounted for 28.9%.^[5]^ In the meanwhile, as a sudden and pandemic infectious disease, COVID-19 causes extreme fear and uncertain emotional reactions in the public. It often leads to negative social behavior, which may involve anxiety, depression, insomnia, aggression, depression and other public mental health problems.^[6]^ As a consequence, how to effectively control the rapid spread of COVID-19 and how to effectively treat the rising number of diagnosed patients has become an urgent worldwide problem to be solved.

Xuanfei Baidu granule (XFBD) is one of the 3 preferred prescriptions in the “COVID-19 diagnosis and treatment Plan (eighth Edition)” issued by the National Health Commission of China. It was jointly formulated by Zhang Boli, academician of the Chinese Academy of Engineering, and Professor Liu Qingquan, dean of Beijing traditional Chinese Medicine Hospital affiliated to Capital Medical University, in the first line of anti-epidemic, for the treatment of mild and ordinary COVID-19 patients.^[7]^ The prescription, with Ma Xing Shigan decoction as the core, is composed of raw ephedra 6 g, bitter almond 15 g, raw gypsum 30 g, raw almond 30 g, raw plaster 30 g, Atractylodes macrocephala 10 g, patchouli 15 g, artemisia grass 12 g, Polygonum cuspidatum 20 g, verbena 30 g, dried Reed root 30 g, Fructus thunbergii 15 g, tangerine red 15 g, raw licorice 10 g.^[8]^ Adding Atractylodes macrocephala and patchouli to help remove dampness, Polygonum cuspidatum clearing heat and detoxification, verbena activating blood circulation and dredging collaterals are used together to play the effect of dispelling lung dampness, clearing heat and penetrating evil, purging lung and detoxification,^[9]^ XFBD has played a significant effect in the clinical treatment of the first line of epidemic prevention and control in Wuhan. In the meanwhile, pharmacological studies on XFBD show that XFBD has a regulatory effect on inflammatory factors such as IL6, chemokine CXCL8, and related T cells (Th17, Th1, Th2).^[10]^ It contributes to inhibit the storm of inflammatory factors and overactivated immune response caused by 2019-nCoV infection.^[11]^ It is crucial for the early regulation of inflammation induced by virus infection. XFBD's proprietary Chinese medicine preparations have been approved for sale in China in March 2021.

XFBD combined with conventional antiviral drugs in the treatment of COVID-19 has been widely used in clinic. However, it is lack of systematic evaluation and other evidence-based medical evidence to support because most of the current studies on XFBD are the latest single center, small sample clinical randomized controlled trials. As a consequence, this scheme through the collation of literature for systematic evaluation and meta-analysis, to evaluate the efficacy and safety of XFBD in the treatment of COVID-19 patients, providing more comprehensive evidence for the large-scale clinical application of XFBD.

## 2 Methods

### 2.1 Inclusion criteria

#### 2.1.1 Design type

Randomized controlled trials.

#### 2.1.2 Objects

The subjects were diagnosed or suspected patients with COVID-19, and their diagnostic criteria met the definition of “COVID-19 diagnosis and treatment Plan (eighth Edition)”. The diagnostic criteria are as follows:

##### 2.1.2.1 Suspected case

There is any 1 item in the history of epidemiology and conforms to any 2 items of the clinical manifestations according to the comprehensive analysis of the following epidemiological history and clinical manifestations; If there is no clear history of epidemiology, it conforms to any 2 items of the clinical manifestations, and novel coronavirus's specific IgM antibody is positive, or 3 items of the clinical manifestations.

###### 2.1.2.1.1 History of epidemiology

The travel or residence history of the reported community within 14 days before the onset of the disease; Have a history of contact with novel coronavirus infected patients or asymptomatic infected persons within 14 days before the onset of the disease; Have contact with patients with fever or respiratory symptoms from the case reporting community within 14 days before the onset of the disease; Cluster onset (2 or more cases of fever and/or respiratory symptoms occurred within 2 weeks in small areas such as family, office, school, class, etc).

###### 2.1.2.1.2 Clinical manifestations

COVID-19-related clinical manifestations such as fever and/or respiratory symptoms; The imaging features of COVID-19 mentioned above; The total leukocyte count was normal or decreased and the lymphocyte count was normal or decreased in the early stage of the disease.

##### 2.1.2.2 Confirmed case

Patients who meet the conditions of a suspected case and have one of the following etiological or serological evidence:

Novel coronavirus nucleic acid positive was detected by real-time fluorescence RT-PCR; Viral gene sequencing showed high homology with the known novel coronavirus; Novel coronavirus specific IgM antibody and IgG antibody were positive; Novel coronavirus specific IgG antibody changed from negative to positive, or the titer of IgG antibody in convalescent stage was 4 times or more higher than that in acute stage.

#### 2.1.3 Intervention measures

XFBD+ routine antiviral drugs were used in the test group and conventional antiviral drugs were used in the control group.

#### 2.1.4 Observation index

Effectiveness indicators: nucleic acid test negative rate, lung computed tomography results improvement rate, symptom improvement rate (fever, cough), the incidence of adverse reactions.

### 2.2 Exclusion criteria

All uncontrolled trials and non-randomized controlled trials; historical controls (comparison of the results of studies conducted in 2 different periods); Comparison between disease and non-disease groups; Trials distributed according to patient characteristics (sex, age, disease severity, different etiology, regional distribution, etc.); The control group was treated with other clinical trials of non-western medicine, such as other types of traditional Chinese medicine or proprietary Chinese medicine; Clinical trials with irregular evaluation indexes or without detailed publication of treatment results, clinical trials without statistical basic data; Reviews, animal experiments, special reports of adverse reactions and non-clinical efficacy studies such as pharmacology and pharmacokinetics; Obvious errors or defects in trial design.

### 2.3 Data source and retrieval

The data were collected from the clinical research literature on the treatment of COVID-19 with XFBD published in biomedical journals from 1919 to 2021. PubMed, CochraneLibrary, Web of Science, excerpt database, China National knowledge Infrastructure, Chinese Science and Technology Journal Database, WanFang, etc. Were selected to screen, the WHO International Clinical Laboratory trial Registration platform (apps.who.int/ trial search/) and clinical trials (www. Clinical trials. Gov) ongoing research was also searched. References that met the inclusion criteria of the study would be reviewed one by one to avoid omissions.

The Chinese database was searched by the combination of title or key words and subject words, and the key words were as follows: The key words in English database were as follows: The subject words combined with free words were used for retrieval, the specific retrieval was as follows (Table 1)

**Table 1:** Search strategy for PubMed.

Number	Search terms
1	#1 Search (“COVID 19”[Mesh]) OR (“COVID-19 Virus Disease”[Mesh]) OR (“COVID 19 Virus Disease” [Mesh]) OR (“Blood Pressures, High”[Mesh]) OR (“High Blood Pressure”[Mesh]) OR (”COVID-19 Virus Diseases“ [Mesh]) OR (”Disease, COVID-19 Virus“ [Mesh]) OR (”Virus Disease, COVID-19“ [Mesh]) OR (”COVID-19 Virus Infection“ [Mesh]) OR (”COVID 19 Virus Infection“ [Mesh]) OR (”COVID-19 Virus Infections“ [Mesh]) OR (”Infection, COVID-19 Virus“ [Mesh]) OR (”Virus Infection, COVID-19“ [Mesh]) OR (”2019-nCoV Infection“ [Mesh]) OR (”2019 nCoV Infection“ [Mesh]) OR (”2019-nCoV Infections“ [Mesh]) OR (”Infection, 2019-nCoV“ [Mesh]) OR (”Coronavirus Disease-19“ [Mesh]) OR (”Coronavirus Disease 19“ [Mesh]) OR (”Coronavirus Disease-19“ [Mesh]) OR (”Coronavirus Disease 19“ [Mesh]) OR (”2019 Novel Coronavirus Disease“ [Mesh]) OR (”2019 Novel Coronavirus Infection“ [Mesh]) OR (”2019-nCoV Disease“ [Mesh]) OR (”2019-nCoV Diseases“ [Mesh]) OR (”Disease, 2019-nCoV“ [Mesh]) OR (”Coronavirus Disease 2019“ [Mesh]) OR (”Disease 2019, Coronavirus“ [Mesh]) OR (”SARS Coronavirus 2 Infection“ [Mesh]) OR (”SARS-CoV-2 Infection“ [Mesh]) OR (”SARS-CoV-2 Infections“ [Mesh]) OR (”COVID-19 Pandemic“ [Mesh]) OR (”COVID 19 Pandemic“ [Mesh]) OR (”COVID-19 Pandemics“ [Mesh]) OR (”Pandemic, COVID-19" [Mesh])
2	#2 Search (“Xuanfei-Baidu granules”) OR (“Xuanfei Baidu Decoction”) OR (“Xuanfei Baidu”)
3	#3 Search(“randomized controlled trial” [Title/Abstract]) OR (“randomized”[Title/Abstract]) OR (“randomly”[Title/Abstract])
4	#1 AND #2 AND #3

### 2.4 Data collection and analysis

#### 2.4.1 Literature screening

**Figure 1:**
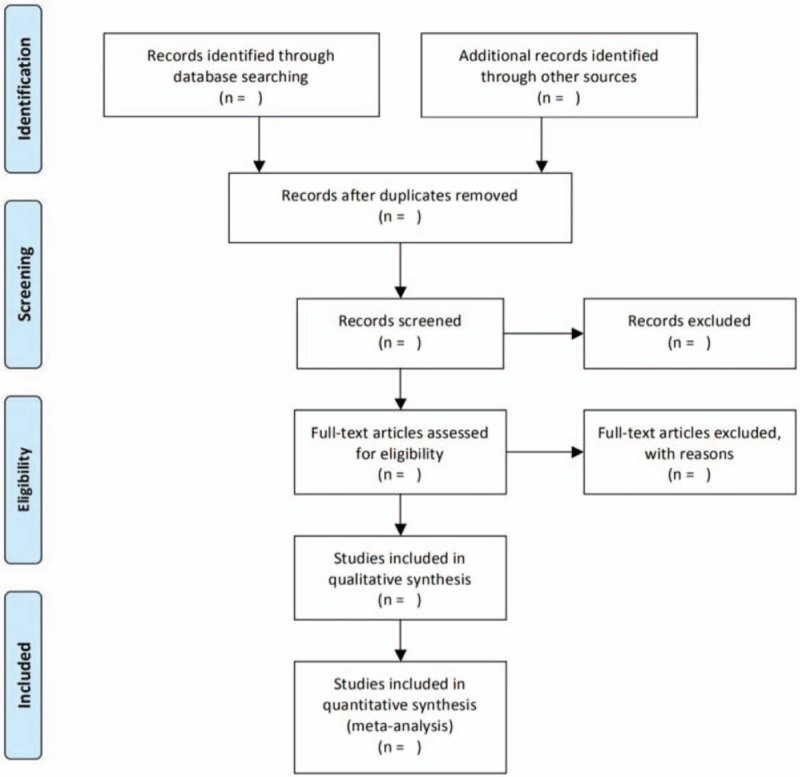
PRISMA flowchart.

Two researchers independently screened the literature, extracted data and quality evaluation and cross-checked, and in case of differences, solicit the opinions of the third party according to the established screening criteria. First of all, researchers read the title and abstract of the literature for preliminary screening, excluded the literature that obviously did not meet the inclusion criteria, and further read the full text for rescreening to determine whether it was included or not. The literature screening process was given in the (PRISMA) flowchart of “preferred report items for systematic review and meta-analysis” (Fig. 1).

#### 2.4.2 Extraction result

The researchers used Excel to establish a data extraction table, which included: Basic information of the literature: title, author, journal published, year; Basic situation of study subjects, grouping, course of disease, baseline, diagnosis and inclusion/exclusion criteria, randomized and blind use; Intervention measures, course of treatment; Elements of risk bias assessment; Outcome indicators: including effectiveness, safety, etc.

If complete information is not available, we would contact the author by email. If the complete data are still not available, the literature would be deleted.

#### 2.4.3 Methodological quality evaluation

The bias risk assessment tool recommended by Cochrane system evaluator manual 5.3.0 and the improved Jadad score scale were used to evaluate the quality of the study. The bias risk assessment tool recommended by Cochrane system evaluator manual 5.3.0 was used to evaluate the study quality according to 7 aspects: random method, assignment concealment, subject blind method, blind result evaluation method, data integrity, selective report and other bias.

#### 2.4.4 Data analysis

The collected information was statistically analyzed by RevMan 5.3.0 software. The counting data were analyzed by relative risk or odds ratio. In the analysis of measurement data, if the measured value of the outcome was based on the meandifference, mean difference analysis obtained by the same measurement unit, and if the same outcome was evaluated but measured according to different methods, the standardized mean difference analysis was used. In addition, the composite results were expressed by the effect value and its 95% confidence interval.

#### 2.4.5 Heterogeneity evaluation

The clinical heterogeneity and statistical heterogeneity between studies were judged. Clinical heterogeneity was judged according to the similarity of research objects, intervention measures, control and outcome indicators between studies; statistical heterogeneity was evaluated by *I*^2^: If *I*^2^ ≤ 50% and *P* > .1, statistical homogeneity was considered to be good. Fixed effect model was used to merge. If *I*^2^ > 50% or *P* ≤ .1, the statistical heterogeneity was large, then the source of heterogeneity was further analyzed. After the obvious clinical heterogeneity was excluded, random effect model was used for Meta analysis. When there was obvious clinical heterogeneity, it should be treated by subgroup analysis or sensitivity analysis, or only descriptive analysis.

#### 2.4.6 Publication bias

If a certain outcome index was included in more than 10 articles, the funnel chart and Egger test should be used to analyze whether there was publication bias.

#### 2.4.7 Sensitivity analysis

Sensitivity analysis was carried out by using “leave-one-out” to determine whether the results were affected by different analysis methods (random effect model or fixed effect model), and to prove the robustness of the results.

#### 2.4.8 GRADE evidence quality classification^[12]^

For the results of this systematic evaluation, the evidence quality evaluation system GRADE was used to evaluate the evidence quality. The evidence quality was divided into high quality, medium quality, low quality and very low quality. In addition, the recommended grade was divided into strong and weak levels.

## 3 Conclusion

This study intends to evaluate the efficacy and safety of XFBD in the treatment of COVID-19 patients. COVID-19 belongs to the category of “plague” in traditional Chinese medicine,^[13]^ and traditional Chinese medicine often has a unique effect on the treatment of “plague”. XFBD has a definite curative effect in the clinical practice of treating COVID-19, especially in reducing the conversion of mild patients to severe diseases.^[14]^ This study provides evidence of evidence-based medicine for the efficacy and safety of XFBD in the treatment of COVID-19. In addition, it provides a new choice for a variety of treatment options for COVID-19.

The limitations of the study are as follows:

1. The sample size of randomized controlled trials literature on both is small, or the epidemic reasons can not strictly guarantee the test process because the related studies on XFBD and COVID-19 are more preface, which may affect the reliability of the results;
2. The research method of this study has some shortcomings, which reduces the quality and authenticity of the test.

It is necessary to pay more attention to expand the scope of literature retrieval, include more high-quality literature, pay more attention to the rigor and standardization of test design and test methods, and strive to provide higher quality evidence for the conclusion in the follow-up research.

## Author contributions

**Conceptualization:** Jisen Zhao, Yongcheng Liu.

**Data curation:** Jisen Zhao, Dong Guo, Maoxia Fan.

**Formal analysis:** Jisen Zhao, Maoxia Fan.

**Funding acquisition:** Dong Guo.

**Methodology:** Maoxia Fan, Yongcheng Liu.

**Resources:** Yongcheng Liu.

**Software:** Yongcheng Liu.

**Writing – original draft:** Jisen Zhao, Dong Guo, Maoxia Fan, Yongcheng Liu.

**Writing – review & editing:** Jisen Zhao, Dong Guo, Maoxia Fan.
